# Work-sensitive Dynamic Complexity of Formal Languages

**DOI:** 10.1007/978-3-030-71995-1_25

**Published:** 2021-03-23

**Authors:** Jonas Schmidt, Thomas Schwentick, Till Tantau, Nils Vortmeier, Thomas Zeume

**Affiliations:** 8 University of Oxford, Oxford, UK; 9 Sorbonne Université - LIP6, Paris, France; 10 TU Dortmund University, Dortmund, Germany; 11 Universität zu Lübeck, Lübeck, Germany; 12 University of Zurich, Zurich, Switzerland; 13 Ruhr University Bochum, Bochum, Germany

**Keywords:** Dynamic complexity, work, parallel constant time.

## Abstract

Which amount of parallel resources is needed for updating a query result after changing an input? In this work we study the amount of work required for dynamically answering membership and range queries for formal languages in parallel constant time with polynomially many processors. As a prerequisite, we propose a framework for specifying dynamic, parallel, constant-time programs that require small amounts of work. This framework is based on the dynamic descriptive complexity framework by Patnaik and Immerman.

### 1 Introduction

Which amount of parallel resources is needed for updating a query result after changing an input, in particular if we only want to spend constant parallel time?

In classical, non-dynamic computations, parallel constant time is well understood. Constant time on CRAMs, a variant of CRCW-PRAMs used by Immerman [15], corresponds to constant-depth in circuits, so, to the circuit class $\mathsf{AC}^0$, as well as to expressibility in first-order logic with built-in arithmetic (see, for instance, the books of Immerman [15, Theorem 5.2] and Vollmer [26, Theorems 4.69 and 4.73]). Even more, the amount of work, that is, the overall number of operations of all processors, is connected to the number of variables required by a first-order formula [15, Theorem 5.10].

However, the work aspect of constant parallel time algorithms is less understood for scenarios where the input is subject to changes. To the best of our knowledge, there is only little previous work on constant-time PRAMs in dynamic scenarios. A notable exception is early work showing that spanning trees

* A full version of the paper is available at [21], https://arxiv.org/abs/2101.08735

and connected components can be computed in constant time by CRCW-PRAMs with $O(n^4)$ and $O(n^2)$ processors, respectively [24].

In an orthogonal line of research, parallel dynamic constant time has been studied from a logical perspective in the dynamic complexity framework by Patnaik and Immerman [20] and Dong, Su, and Topor [7,6]. In this framework, the update of query results after a change is expressed by first-order formulas. The formulas may refer to auxiliary relations, whose updates in turn are also specified by first-order formulas (see Section 3 for more details). The queries maintainable in this fashion constitute the dynamic complexity class DynFO. Such queries can be updated by PRAMs in constant time with a polynomial number of processors. In this line of work, the main focus in recent years has been on proving that queries are in DynFO, and thus emphasised the constant time aspect. It has, for instance, been shown that all context-free languages [11] and the reachability query [5] are in DynFO.

However, if one tries to make the "DynFO approach" for dynamic problems relevant for practical considerations, the work that is needed to carry out the specified updates, hence the *work* of a parallel algorithm implementing them, is a crucial factor. The current general polynomial upper bounds are too coarse. In this paper, we therefore initiate the investigation of more work-efficient dynamic programs that can be specified by first-order logic and that can therefore be carried out by PRAMs in constant time. To do so, we propose a framework for specifying such dynamic, parallel, constant-time programs, which is based on the DynFO framework, but allows for more precise (and better) bounds on the necessary work of a program.

**Goal 1.1.** *Extend the formal framework of dynamic complexity towards the consideration of parallel work.*

Towards this goal, we link the framework we propose to the CRAM framework in Section 3. In fact, the new framework also takes a somewhat wider perspective, since it does not focus exclusively at one query under a set of change operations, but rather considers dynamic problems that may have several change and query operations (and could even have operations that combine the two). Therefore, from now on we speak about dynamic problems and not about (single) queries.

**Goal 1.2.** *Find work-efficient* DynFO*-programs for dynamic problems that are known to be in* DynFO *(but whose dynamic programs*[5] *are not competitive, work-wise).*

Ideally we aim at showing that dynamic problems can be maintained in DynFO with sublinear or even polylogarithmic work. One line of attack for this goal is to study dynamic algorithms and to see whether they can be transformed into parallel $\mathcal{O}(1)$-time algorithms with small work. There is a plethora of work

[5] In the field of dynamic complexity the term "dynamic program" is traditionally used for the programs for updating the auxiliary data after a change. The term should not be confused with the "dynamic programming" technique used in algorithm design.

that achieves polylogarithmic sequential update time (even though, sometimes only amortised), see for instance [3,9,12,13]. For many of these problems, it is known that they can be maintained in constant parallel time with polynomial work, e.g. as mentioned above, it has been shown that connectivity and maintenance of regular (and even context-free) languages is in DynFO.

In this paper, we follow this approach for dynamic string problems, more specifically, dynamic problems that allow membership and range queries for regular and context-free languages. Our results can be summarised as follows.

We show in Section 5 that regular languages can be maintained in constant time with $\mathcal{O}(n^\epsilon)$ work for all $\epsilon > 0$ and that for star-free languages even work $\mathcal{O}(\log n)$ can be achieved. These results hold for range and membership queries.

For context-free languages, the situation is not as nice, as we observe in Section 6. We show that subject to a well-known conjecture, we cannot hope for maintaining membership in general context-free languages in DynFO with less than $\mathcal{O}(n^{1.37-\epsilon})$ work. The same statement holds even for the bound $\mathcal{O}(n^{2-\epsilon})$ and "combinatorial dynamic programs". For Dyck languages, that is, sets of well-formed strings of parentheses, we show that this barrier does not apply. Their membership problem can be maintained with $\mathcal{O}(n(\log n)^3)$ work in general, and with polylogarithmic work if there is only one kind of parentheses. By a different approach, range queries can be maintained with work $\mathcal{O}(n^{1+\epsilon})$ in general, and $\mathcal{O}(n^\epsilon)$ for one parenthesis type.

*Related work.* A complexity theory of incremental time has been developed in [19]. We discuss previous work on dynamic complexity of formal languages in Sections 5 and 6.

### 2 Preliminaries

Since dynamic programs are based on first-order logic, we represent inputs like graphs and strings as well as "internal" data structures as logical structures.

A *schema* $\tau$ consists of a set of relation symbols and function symbols with a corresponding arity. A constant symbol is a function symbol with arity 0. A *structure* $\mathcal{D}$ over schema $\tau$ with finite domain $D$ has, for every $k$-ary relation symbol $R \in \tau$, a relation $R^\mathcal{D} \subseteq D^k$, as well as a function $f^\mathcal{D} \colon D^k \to D$ for every $k$-ary function symbol $f \in \tau$. We allow partially defined functions and write $f^\mathcal{D}(\bar{a}) = \bot$ if $f^\mathcal{D}$ is not defined for $\bar{a}$ in $\mathcal{D}$. Formally, this can be realized using an additional relation that contains the domain of $f^\mathcal{D}$. We occasionally also use functions $f^\mathcal{D} \colon D^k \to D^\ell$ for some $\ell > 1$. Formally, such a function represents $\ell$ functions $f_1^\mathcal{D}, \dots, f_\ell^\mathcal{D} \colon D^k \to D$ with $f^\mathcal{D}(\bar{a}) \stackrel{\text{def}}{=} (f_1^\mathcal{D}(\bar{a}), \dots, f_\ell^\mathcal{D}(\bar{a}))$.

Throughout this work, the structures we consider provide a linear order $\leq$ on their domain $D$. As we can thus identify $D$ with an initial sequence of the natural numbers, we usually just assume that $D = [n] \stackrel{\text{def}}{=} \{0, \dots, n-1\}$ for some natural number $n$.

We assume familiarity with first-order logic FO, and refer to [17] for basics of Finite Model Theory. In this paper, unless stated otherwise, first-order formulas

*always* have access to a linear order on the domain, as well as compatible functions $+$ and $\times$ that express addition and multiplication, respectively. This holds in particular for formulas in dynamic programs. We use the following "if-then-else" construct: if $\varphi$ is a formula, and $t_1$ and $t_2$ are terms, then $\text{ITE}(\varphi, t_1, t_2)$ is a term. Such a term evaluates to the result of $t_1$ if $\varphi$ is satisfied, otherwise to $t_2$.

Following [11], we encode words of length (at most) $n$ over an alphabet $\Sigma$ by *word structures*, that is, as relational structures $W$ with universe $\{0, \dots, n-1\}$, one unary relation $R_\sigma$ for each symbol $\sigma \in \Sigma$ and the canonical linear order $\leq$ on $\{0, \dots, n-1\}$. We only consider structures for which, for every position $i$, $R_\sigma(i)$ holds for at most one $\sigma \in \Sigma$ and write $W(i) = \sigma$ if $R_\sigma(i)$ holds and $W(i) = \epsilon$ if no such $\sigma$ exists. We write $\text{word}(W)$ for the word represented by $W$, that is, the concatenation $w = W(0) \circ \dots \circ W(n-1)$. As an example, the word structure $W_0$ with domain $\{0, 1, 2, 3\}$, $W(1) = a$, $W(3) = b$ and $W(0) = W(2) = \epsilon$ represents the string $ab$. We write $\text{word}(W)[\ell, r]$ for the word $W(\ell) \circ \dots \circ W(r)$.

Informally, a *dynamic problem* can be seen as a data type: it consists of some underlying structure together with a set $\Delta$ of operations. We distinguish between *change operations* that can modify the structure and *query operations* that yield information about the structure, but combined operations could be allowed, as well. Thus, a dynamic problem is characterised by the schema of its underlying structures and the operations that it supports.[6]

In this paper, we are particularly interested in dynamic language problems, defined as follows. Words are represented as word structures $W$ with elementary change operations $\text{SET}_\sigma(i)$ (with the effect that $W(i)$ becomes $\sigma$ if it was $\epsilon$ before) and $\text{RESET}(i)$ (with the effect that $W(i)$ becomes $\epsilon$).

For some fixed language $L$ over some alphabet $\Sigma$, the dynamic problem $\text{RANGEMEMBER}(L)$ further supports one query operation $\text{RANGE}(\ell, r)$. It yields the result true, if $\text{word}(W)[\ell, r]$ is in $L$, and otherwise false.

In the following, we denote a word structure $W$ as a sequence $w_0 \dots w_{n-1}$ of letters with $w_i \in \Sigma \cup \{\epsilon\}$ in order to have an easier, less formal notation. Altogether, the dynamic problem $\text{RANGEMEMBER}(L)$ is defined as follows.

**Problem:** $\text{RANGEMEMBER}(L)$
**Input:** A sequence $w = w_0 \dots w_{n-1}$ of letters with $w_i \in \Sigma \cup \{\epsilon\}$
**Changes:** $\text{SET}_\sigma(i)$ for $\sigma \in \Sigma$: Sets $w_i$ to $\sigma$, if $w_i = \epsilon$
$\text{RESET}(i)$: Sets $w_i$ to $\epsilon$
**Queries:** $\text{RANGE}(\ell, r)$: Is $w_\ell \circ \cdots \circ w_r \in L$?

In this example, the query RANGE maps (binary) pairs of domain elements to a truth value and thus defines a (binary) relation over the universe of the input word structure. We call such a query *relational.* We will also consider *functional* queries mapping tuples of elements to elements.

Another dynamic problem considered here is $\text{MEMBER}(L)$ which is defined similarly as $\text{RANGEMEMBER}(L)$ but instead of RANGE only has the Boolean query operation MEMBER that yields true if $w_0 \circ \dots \circ w_{n-1} \in L$ holds.

[6] This view is a bit broader than the traditional setting of Dynamic Complexity, where there can be various change operations but usually only one fixed query is supported.

### 3 Work-sensitive Dynamic Complexity

Since we are interested in the work that a dynamic program does, our specification mechanism for dynamic programs is considerably more elaborated than the one used in previous papers on dynamic complexity. We introduce the mechanism in this section in two steps. First the general form of dynamic programs and then a more pseudo-code oriented syntax. Afterwards, we discuss how these dynamic programs translate into work-efficient constant-time parallel programs.

#### 3.1 The Dynamic Complexity Framework

Our general form of dynamic programs mainly follows [23], but is adapted to the slightly broader view of a dynamic problem as a data type. For a more gentle introduction to dynamic complexity, we refer to [22].

The goal of a *dynamic program* for a dynamic problem $\Pi$ is to support all its operations $\Delta$. To do so, it stores and updates an auxiliary structure $\mathcal{A}$ over some schema $\tau_{\text{aux}}$, over the same domain as the input structure $\mathcal{I}$ for $\Pi$.

A (first-order) dynamic program $\mathcal{P}$ consists of a set of (first-order) *update rules* for change operations and *query rules* for query operations. More precisely, a program has one query rule over schema $\tau_{\text{aux}}$ per query operation that specifies how the (relational) result of that operation is obtained from the auxiliary structure. Furthermore, for each change operation $\delta \in \Delta$, it has one update rule per auxiliary relation or function that specifies the updates after a change based on $\delta$.

A query rule is of the form **on query** $Q(\bar{p})$ **yield** $\varphi_Q(\bar{p})$, where $\varphi_Q$ is the (first-order) *query formula* with free variables from $\bar{p}$.

An update rule for a $k$-ary auxiliary relation $R$ is of the form

$$\textbf{on change } \delta(\bar{p}) \textbf{ update } R \textbf{ at } (t_1(\bar{p};\bar{x}),\ldots,t_k(\bar{p};\bar{x})) \textbf{ as } \varphi_\delta^R(\bar{p};\bar{x}) \textbf{ where } C(\bar{x}).$$

Here, $\varphi_\delta^R$ is the (first-order) *update formula*, $t_1,\ldots,t_k$ are first-order terms (possibly using the ITE construct) over $\tau_{\text{aux}}$, and $C(\bar{x})$, called a *constraint* for the tuple $\bar{x} = x_1,\ldots,x_\ell$ of variables, is a conjunction of inequalities $x_i \leq f_i(n)$ using functions $f_i\colon \mathbb{N} \to \mathbb{N}$, where $n$ is the size of the domain and $1 \leq i \leq \ell$. We demand that all functions $f_i$ are first-order definable from $+$ and $\times$.

The effect of such an update rule after a change operation $\delta(\bar{a})$ is as follows: the new relation $R^{\mathcal{A}'}$ in the updated auxiliary structure $\mathcal{A}'$ contains all tuples from $R^{\mathcal{A}}$ that are *not* equal to $(t_1(\bar{a};\bar{b}),\ldots,t_k(\bar{a};\bar{b}))$ for any tuple $\bar{b}$ that satisfies the constraints $C$; and additionally $R^{\mathcal{A}'}$ contains all tuples $(t_1(\bar{a};\bar{b}),\ldots,t_k(\bar{a};\bar{b}))$ such that $\bar{b}$ satisfies $C$ and $\mathcal{A} \models \varphi_\delta^R(\bar{a};\bar{b})$ holds.

Phrased more operationally, an update is performed by enumerating all tuples $\bar{b}$ that satisfy $C$, evaluating $\varphi_\delta^R(\bar{a};\bar{b})$ on the old auxiliary structure $\mathcal{A}$, and depending on the result adding the tuple $(t_1(\bar{a};\bar{b}),\ldots,t_k(\bar{a};\bar{b}))$ to $R$ (if it was not already present), or removing that tuple from $R$ (if it was present).

Update rules for auxiliary functions are similar, but instead of an update formula that decides whether a tuple of the form $(t_1(\bar{a};\bar{b}),\ldots,t_k(\bar{a};\bar{b}))$ is contained in the updated relation, it features an update term that determines the new function value for a function argument of the form $(t_1(\bar{a};\bar{b}),\ldots,t_k(\bar{a};\bar{b}))$.

We say that $\mathcal{P}$ is a dynamic program for a dynamic problem $\Pi$ if it supports all its operations and, in particular, always yields correct results for query operations. More precisely, if the result of applying a query operation after a sequence $\alpha$ of change operations on an initial structure $\mathcal{I}_0$ yields the same result as the evaluation of the query rule on the auxiliary structure that is obtained by applying the update rules corresponding to the change operations in $\alpha$ to an initial auxiliary structure $\mathcal{A}_0$. Here, an initial input structure $\mathcal{I}_0$ over some domain $D$ is *empty*, that is, it is a structure with empty relations and with all function values being undefined ($\perp$). The initial auxiliary structure $\mathcal{A}_0$ is over the same domain $D$ as $\mathcal{I}_0$ and is defined from $\mathcal{I}_0$ by some FO-definable initialization.

By DynFO, we denote the class of all dynamic problems that have a dynamic program in the sense we just defined.

#### 3.2 A syntax for work-efficient dynamic programs

In this paper we are particularly interested in dynamic programs that require little work to update the auxiliary structure after every change operation and to compute the result of a query operation. However, since dynamic programs do not come with an execution model, there is no direct way to define, say, when a DynFO-programs has polylogarithmic-work, syntactically.

We follow a pragmatic approach here. We define a pseudo-code-based syntax for *update* and *query procedures* that will be used in place of the update and query *formulas* in rules of dynamic programs. This syntax has three important properties: (1) it is reasonably well readable (as opposed to strict first-order logic formulas), (2) it allows a straightforward translation of rules into proper DynFO-programs, and (3) it allows to associate a "work-bounding function" to each rule and to translate it into a PRAM program with $\mathcal{O}(1)$ parallel time and work bounded by this function.

The syntax of the pseudo-code has similarities with Abstract State Machines [4] and the PRAM-syntax of [16]. For simplicity, we describe a minimal set of syntactic elements that suffice for the dynamic programs in this paper. We encourage readers to have a look at Section 4 for examples of update rules with pseudo-code syntax.

We only spell out a syntax for *update procedures* that can be used in place of the update formula $\varphi_\delta^R(\bar{p};\bar{x})$ of an update rule

**on change** $\delta(\bar{p})$ **update** $R$ **at** $(t_1(\bar{p};\bar{x}),\ldots,t_k(\bar{p};\bar{x}))$ **as** $\varphi_\delta^R(\bar{p};\bar{x})$ **where** $C(\bar{x})$.

Query procedures are defined similarly, but they can not invoke any change operations for supplementary instances, and their only free variables are from $\bar{p}$.

We allow some compositionality: a dynamic program on some *main instance* can use *supplementary instances* of other dynamic problems and invoke change or query operations of other dynamic programs on those instances. These supplementary instances are declared on a global level of the dynamic program and each has an associated identifier.

Update procedures $P = P_1; P_2$ consist of two parts. In the *initial procedure* $P_1$ no reference to the free variables from $\bar{x}$ are allowed, but change operations for supplementary instances can be invoked. We require that, for each change operation $\delta$ of the main instance and each supplementary instance $\mathcal{S}$, at most one update rule for $\delta$ invokes change operations for $\mathcal{S}$.

In the *main procedure* $P_2$, no change operations for supplementary instances can be invoked, but references to $\bar{x}$ are allowed.

More precisely, both $P_1$ and $P_2$ can use (a series of) instructions of the following forms:

- assignments $f(\bar{y}) \leftarrow$ `term` of a function value,
- assignments $R(\bar{y}) \leftarrow$ `condition` of a Boolean value,
- conditional branches **if** `condition` **then** $P'$ **else** $P''$, and
- parallel branches **for** $z \leq g(n)$ **pardo** $P'$.

Semantically, here and in the following $n$ always refers to the size of the domain of the main instance. The initial procedure $P_1$ can further use change invocations `instance`.$\delta(\bar{y})$. However, they are not allowed in the scope of parallel branches. And we recall that in $P_1$ no variables from $\bar{x}$ can be used.

The main procedure $P_2$ can further use return statements **return** `condition` or **return** `term`, but not inside parallel branches.

Of course, initial procedures can only have initial procedures $P'$ and $P''$ in conditional and parallel branches, and analogously for main procedures.

Conditions and terms are defined as follows. In all cases, $\bar{y}$ denotes a tuple of terms and $z$ is a *local variable*, not occurring in $\bar{p}$ or $\bar{x}$. In general, a *term* evaluates to a domain element (or to $\perp$). It is built from

- local variables and variables from $\bar{p}$ and $\bar{x}$,
- function symbols from $\tau_{\text{aux}}$ and previous function assignments,
- if-then-else terms **if** `condition` **then** `term`$'$ **else** `term`$''$,
- functional queries `instance`.$Q(\bar{y})$, and
- expressions **getUnique**$(z \leq g(n) \mid$ `condition`$)$.

For the latter expression it is required that there is always exactly one domain element $a \leq g(n)$ satisfying `condition`.

A *condition* evaluates to `true` or `false`. It may be

- an atomic formula with relation symbols from $\tau_{\text{aux}}$ or previous assignments, with terms as above,
- an expression **exists**$(z \leq g(n) \mid$ `condition`$)$,
- a relational query `instance`.$Q(\bar{y})$ with terms $\bar{y}$, and
- a Boolean combination of conditions.

All functions $g\colon \mathbb{N} \to \mathbb{N}$ in these definitions are required to be $\mathsf{FO}$-definable. For assignments of relations $R$ and functions $f$ we demand that these symbols do *not* appear in $\tau_{\text{aux}}$. If an assignment with a head $f(\bar{y})$ or $R(\bar{y})$ occurs in the scope of a parallel branch that binds variable $z$, then $z$ has to occur as a term $y_i$

in $\bar{y}$. We further demand that update procedures are well-formed, in the sense that every execution path ends with a return statement of appropriate type.

In our pseudo-code algorithms, we display update procedures $P = P_1; P_2$ with initial procedure $P_1$ and main procedure $P_2$ as

**on change** $\delta(\bar{p})$ **with** $P_1$
**update** $R$ **at** $(t_1(\bar{p}, \bar{x}), \ldots, t_k(\bar{p}, \bar{x}))$, **for all** $C(\bar{x})$, **by**: $P_2$.

to emphasise that $P_1$ only needs to be evaluated once for the update of $R$, and not once for every different value of $\bar{x}$.

In a nutshell, the semantics of an update rule

**on change** $\delta(\bar{p})$ **update** $R$ **at** $(t_1(\bar{p}; \bar{x}), \ldots, t_k(\bar{p}; \bar{x}))$ **as** $P$ **where** $C(\bar{x})$

is defined as in Subsection 3.1, but $\mathcal{A} \models \varphi_\delta^R(\bar{a}, \bar{b})$ has to be replaced by the condition that $P$ returns true under the assignment $(\bar{p} \mapsto \bar{a}; \bar{x} \mapsto \bar{b})$.

For update rules for auxiliary functions, $P$ returns the new function value instead of a Boolean value.

Since $P_1$ is independent of $\bar{x}$, in the semantics, it is only evaluated once. In particular, any change invocations are triggered only once.

With Procedural-DynFO-programs we refer to the above class of dynamic update programs. Here and later we will introduce abbreviations as syntactic sugar, for example the sequential loop **for** $z \leq m$ **do** $P$, where $m \in \mathbb{N}$ needs to be a fixed natural number.

We show next that update and query procedures can be translated into constant-time CRAM programs. Since the latter can be translated into FO-formulas [14, Theorem 5.2], therefore Procedural-DynFO-programs can be translated in DynFO-programs.

#### 3.3 Implementing Procedural-DynFO-programs as PRAMs

We use *Parallel Random Access Machines* (PRAMs) as the computational model to measure the work of our dynamic programs. A PRAM consists of a number of processors that work in parallel and use a shared memory. We only consider *CRAMs*, a special case of Concurrent-Read Concurrent-Write model (CRCW PRAM), i.e. processors are allowed to read and write concurrently from and to the same memory location, but if multiple processors concurrently write the same memory location, then all of them need to write the same value. For an input of size $n$ we denote the *time* that a PRAM algorithm needs to compute the solution as $T(n)$. The *work* $W(n)$ of a PRAM algorithm is the sum of the number of all computation steps of all processors made during the computation. For further details we refer to [14,16].

It is easy to see that Procedural-DynFO programs $\mathcal{P}$ can be translated into $\mathcal{O}(1)$-time CRAM-programs $\mathcal{C}$. To be able to make a statement about (an upper bound of) the work of $\mathcal{C}$, in the full version of this paper we associate a function $w$ with update rules and show that every update rule $\pi$ can be implemented by a $\mathcal{O}(1)$-time CRAM-program with work $\mathcal{O}(w)$. Likewise for query rules.

In a nutshell, the work of an update procedure mainly depends on the scopes of the (nested) parallel branches and the amount of work needed to query and update the supplementary instances. The work of a whole update rule is then determined by adding the work of the initial procedure once and adding the work of the main procedure for each tuple that satisfies the constraint of the update rule.

### 4 A simple work-efficient Dynamic Program

In this section we consider a simple dynamic problem with a fairly work-efficient dynamic program. It serves as an example for our framework but will also be used as a subroutine in later sections.

The dynamic problem is to maintain a subset $K$ of an ordered set $D$ of elements under insertion and removal of elements in $K$, allowing for navigation from an element of $D$ to the next larger and smaller element in $K$. That is, we consider the following dynamic problem:

**Problem:** NEXTINK
**Input:** A set $K \subseteq D$ with canonical linear order $\leq$ on $D$
**Changes:** INS($i$): Inserts $i \in D$ into $K$
DEL($i$): Deletes $i \in D$ from $K$
**Queries:** PRED($i$): Returns predecessor of $i$ in $K$, i.e. $\max\{j \in K \mid i > j\}$
SUCC($i$): Returns successor of $i$ in $K$, i.e. $\min\{j \in K \mid i < j\}$

For the smallest (largest) element the result of a PRED (SUCC) query is undefined, i.e. $\perp$. For simplicity, we assume in the following that $D$ is always of the form $[n]$, for some $n \in \mathbb{N}$.

Sequentially, the changes and queries of NEXTINK can be handled in sequential time $\mathcal{O}(\log \log n)$ [9]. Here we show that the problem also has a dynamic program with parallel time $\mathcal{O}(1)$ and work $\mathcal{O}(\log n)$.

**Lemma 4.1.** *There is a* DynFO*-program for* NEXTINK with $\mathcal{O}(\log n)$ work per change and query operation.

*Proof.* The dynamic program uses an ordered binary balanced tree $T$ with leave set $[n]$, and with 0 as its leftmost leaf. Each inner node $v$ represents the interval $S(v)$ of numbers labelling the leafs of the subtree of $v$. To traverse the tree, the dynamic program uses functions 1st and 2nd that map an inner node to its first or second child, respectively, and a function $\text{anc}(v, j)$ that returns the $j$-th ancestor of $v$ in the tree.[7] So, $\text{anc}(v, 2)$ returns the parent of the parent of $v$.

The functions 1st, 2nd and anc are static, that is, they are initialized beforehand and not affected by change operations.

[7] Formally, the $2|D|$ nodes of $T$ can be represented by pairs $(a, b)$ of elements. In our presentation, we disregard these technical issues and use nodes of $T$ just as domain elements.

**Algorithm 1** Querying a successor.

1: **on query** $\textsc{succ}(i)$:
2: **if** $\max(T.\mathrm{root}) \leq i$ **then**
3: **return** $\perp$
4: **else**
5: $k \leftarrow$ **getUnique**$(1 \leq k \leq \log(n) \mid \max(T.\mathrm{anc}(i,k)) > i$
6: $\wedge\ \max(T.\mathrm{anc}(i,k-1)) \leq i$
7: **return** $\min(T.\mathrm{2nd}(T.\mathrm{anc}(i,k)))$

**Algorithm 2** Updating min after an insertion.

1: **on change** $\textsc{ins}(i)$ **update** min **at** $T.\mathrm{anc}(i,k)$, **for all** $k \leq \log n$, **by**:
2: $v \leftarrow T.\mathrm{anc}(i,k)$
3: **if** $\min(v) > i$ **then**
4: **return** $i$
5: **else**
6: **return** $\min(v)$

The idea of the dynamic program is to maintain, for each node $v$, the maximal and minimal element in $K \cap S(v)$ (which is undefined if $K \cap S(v) = \emptyset$), by maintaining two functions min and max. It is easy to see that this information can be updated and queries be answered in $\mathcal{O}(\log n)$ time as the tree has depth $\mathcal{O}(\log n)$. For achieving $\mathcal{O}(\log n)$ work and constant time, we need to have a closer look.

Using min and max, it is easy to determine the $K$-successor of an element $i \in D$: if $v$ is the lowest ancestor of $i$ with $\max(v) > i$, then the $K$-successor of $i$ is $\min(w)$ for the second child $w \stackrel{\text{def}}{=} \mathrm{2nd}(v)$ of $v$. Algorithm 1 shows a query rule for the query operation $\textsc{succ}(i)$. The update of these functions is easy when an element $i$ is inserted into $K$. This is spelled out for min in Algorithm 2. The dynamic program only needs to check if the new element becomes the minimal element in $S(v)$, for every node $v$ that is an ancestor of the leaf $i$.

Algorithm 3 shows how min can be updated if an element $i$ is deleted from $K$: if $i$ is the minimal element of $K$ in $S(v)$, for some node $v$, then $\min(v)$ needs to be replaced by its $K$-successor, assuming it is in $S(v)$.

It is easy to verify the claimed work upper bounds for $\mathcal{P}$. Querying a successor or predecessor via Algorithm 1 needs $\mathcal{O}(\log n)$ work, since Line 6 requires $\mathcal{O}(\log n)$ and all others require $\mathcal{O}(1)$ work. For maintaining the function min the programs in Algorithms 2 and 3 update the value of $\log n$ tuples, but the work per tuple is constant. In the case of a deletion, Line 3 requires $\mathcal{O}(\log n)$ work but is executed only once. The remaining part consists of $\mathcal{O}(\log n)$ parallel executions of statements, each with $\mathcal{O}(1)$ work.

The handling of max and its work analysis is analogous. □

**Algorithm 3** Updating min after a deletion.

**on change** DEL($i$)<br>
    **with**<br>
        $s \leftarrow$ SUCC($i$)<br>
    **update** min **at** $T.\mathrm{anc}(i, k)$, **for all** $k \leq \log n$, **by**:<br>
        $v \leftarrow T.\mathrm{anc}(i, k)$<br>
        **if** $\min(v) \neq i$ **then**<br>
            **return** $\min(v)$<br>
        **else if** $\max(v) = i$ **then**<br>
            **return** $\perp$<br>
        **else**<br>
            **return** $s$

## 5 Regular Languages

In this section, we show that the range problem can be maintained with $o(n)$ work for all regular languages and with polylogarithmic work for star-free languages. For the former we show how to reduce the work of a known DynFO-program. For the latter we translate the idea of [9] for maintaining the range problem for star-free languages in $\mathcal{O}(\log \log n)$ sequential time into a dynamic program with $\mathcal{O}(1)$ parallel time.

### 5.1 DynFO-programs with sublinear work for regular languages

**Theorem 5.1.** *Let $L$ be a regular language. Then* RANGEMEMBER($L$) *can be maintained in DynFO with work $\mathcal{O}(n^\epsilon)$ per query and change operation, for every $\epsilon > 0$.*

The proof of this theorem makes use of the algebraic view of regular languages. For readers not familiar with this view, the basic idea is as follows: for a fixed DFA $\mathcal{A} = (Q, \Sigma, \delta, q_0, F)$, we first associate with each string $w$ a function $f_w$ on $Q$ that is induced by the behaviour of $\mathcal{A}$ on $w$ via $f_w(q) \stackrel{\text{def}}{=} \delta^*(q, w)$, where $\delta^*$ is the extension of the transition function $\delta$ to strings. The set of all functions $f\colon Q \to Q$ with composition as binary operation is a *monoid*, that is, a structure with an associative binary operation $\circ$ and a neutral element, the identity function. Thus, composing the effect of $\mathcal{A}$ on subsequent substrings of a string corresponds to multiplication of the monoid elements associated with these substrings. The *syntactic monoid* $M(L)$ of a regular language $L$ is basically the monoid associated with its minimal automaton.

It is thus clear that, for the dynamic problem RANGEMEMBER($L$) where $L$ is regular, a dynamic program can be easily obtained from a dynamic program for the dynamic problem RANGEEVAL($M(L)$), where RANGEEVAL($M$), for finite monoids $M$, is defined as follows.[8]

[8] We note that, unlike for words, each position always carries a monoid element. However, the empty string of the word case corresponds to the neutral element in

**Problem:** RANGEEVAL($M$)
**Input:** A sequence $m_0 \dots m_{n-1}$ of monoid elements $m_i \in M$
**Changes:** $\text{SET}_m(i)$ for $m \in M$: Replaces $m_i$ by $m$
**Queries:** $\text{RANGE}(\ell, r)$: $m_\ell \circ \dots \circ m_r$

For the proof of Theorem 5.1 we do not need any insights into monoid theory. However, when studying languages definable by first-order formulas in Theorem 5.3 below, we will make use of a known decomposition result.

From the discussion above it is now clear that in order to prove Theorem 5.1, it suffices to prove the following result.

**Proposition 5.2.** *Let $M$ be a finite monoid. For every $\epsilon > 0$,* RANGEEVAL($M$) *can be maintained in DynFO with work $\mathcal{O}(n^\epsilon)$ per query and change operation.*

*Proof sketch.* In [11], it was (implicitly) shown that RANGEMEMBER($L$) is in DynProp (that is, quantifier-free DynFO), for regular languages $L$. The idea was to maintain the effect of a DFA for $L$ on $w[\ell, r]$, for each interval $(\ell, r)$ of positions. This approach can be easily used for RANGEEVAL($M$) as well, but it requires a quadratic number of updates after a change operation, in the worst case.

We adapt this approach and only store the effect of the DFA for $\mathcal{O}(n^\epsilon)$ intervals, by considering a hierarchy of intervals of bounded depth.

The first level in the hierarchy of intervals is obtained by decomposing the input sequence into intervals of length $t$, for a carefully chosen $t$. We call these intervals *base intervals* of height 1 and their subintervals *special intervals* of height 1. The latter are *special* in the sense that they are exactly the intervals for which the dynamic program maintaines the product of monoid elements. In particular, each base interval of height 1 gives rise to $\mathcal{O}(t^2)$ special intervals of height 1. The second level of the hierarchy is obtained by decomposing the sequence of base intervals of height 1 into sequences of length $t$. Each such sequence of length $t$ is combined to one base interval of height 2; and each contiguous subsequence of such a sequence is combined to one special interval of height 2. Again, each base interval of height 2 gives rise to $\mathcal{O}(t^2)$ special intervals of height 2. This process is continued recursively for the higher levels of the hierarchy, until only one base interval of height $h$ remains. We refer to Figure 1 for an illustration of this construction.

The splitting factor $t$ is chosen in dependence of $n$ and $\epsilon$ such that the height of this hierarchy of special intervals only depends on $\epsilon$ and is thus constant for all $n$. More precisely, we fix $\lambda \stackrel{\text{def}}{=} \frac{\epsilon}{2}$ and $t \stackrel{\text{def}}{=} n^\lambda$. Therefore, $h = \log_t(n) = \frac{1}{\lambda}$.

The idea for the dynamic program is to store the product of monoid elements for each special interval. The two crucial observations are then, that (1) the product of each (not necessary special) interval can be computed with the help of a constant number of special intervals, and (2) that each change operation affects at most $t^2$ special intervals per level of the hierarchy and thus at most $ht^2 \in \mathcal{O}(n^\epsilon)$ special intervals in total. We refer to the full version for more details. □

the monoid case. In particular, the initial "empty" sequence consists of $n$ copies of the neutral element.

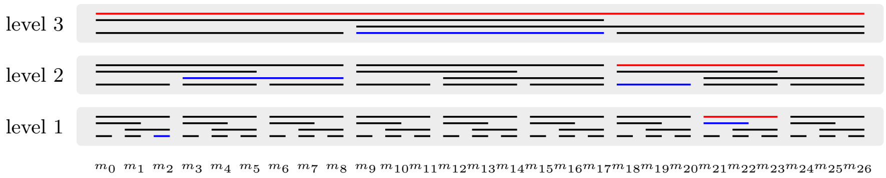

**Fig. 1.** Illustration of special intervals, for $t = 3$. The special intervals of level 3 are $[0,9), [9,18), [18,27), [0,18)$ and $[9,27)$ with base interval $[0,27)$. The result of a query RANGE$(2,22)$ can be computed as $\prod_{i=2}^{22} m_i = \big(m[2,3) \circ m[3,9)\big) \circ m[9,18) \circ \big(m[18,21) \circ m[21,23)\big)$, illustrated above in blue. The affected base intervals for a change at position 23 are marked in red. E.g., the new product $m'[18,27)$ can be computed by $m'[18,27) = m[18,21) \circ m'[21,24) \circ m[24,27)$. As the products are recomputed bottom up, $m'[21,24)$ is already updated.

#### 5.2 DynFO-programs with polylogarithmic work for star-free languages

Although the work bound of Theorem 5.1 for regular languages is strongly sublinear, one might aim for an even more work-efficient dynamic program, especially, since RangeMember$(L)$ can be maintained *sequentially* with logarithmic update time for regular languages [9]. We leave it as an open problem whether for every regular language $L$ there is a DynFO-program for RangeMember$(L)$ with a polylogarithmic work bound. However, we show next that such programs exist for star-free regular languages, in fact they even have a logarithmic work bound. The star-free languages are those that can be expressed by regular expressions that do not use the Kleene star operator but can use complementation.

**Theorem 5.3.** *Let $L$ be a star-free regular language. Then* RangeMember$(L)$ *can be maintained in* DynFO *with work* $\mathcal{O}(\log n)$ *per query and change operation.*

It is well-known that star-free regular languages are just the regular languages that can be defined in first-order logic (without arithmetic!) [18]. Readers might ask why we consider dynamic first-order maintainability of a problem that can actually be *expressed* in first-order logic. The key point is the parallel work here: even though the membership problem for star-free languages can be solved by a parallel algorithm in time $\mathcal{O}(1)$, it inherently requires parallel work $\Omega(n)$.

*Proof sketch.* The proof uses the well-known connection between star-free languages and group-free monoids (see, e.g., [25, Chapter V.3] and [25, Theorem V.3.2]). It thus follows the approach of [9].

In a nutshell, our dynamic program simply implements the algorithms of the proof of Theorem 2.4.2 in [9]. Those algorithms consist of a constantly bounded number of simple operations and a constantly bounded number of searches for a next neighbour in a set. Since the latter can be done in DynFO with work $\mathcal{O}(\log n)$ thanks to Lemma 4.1, we get the desired result for group-free monoids and then for star-free languages. We refer to the full version for more details. □

## 6 Context Free Languages

As we have seen in Section 5, range queries to regular languages can be maintained in DynFO with strongly sublinear work. An immediate question is whether context-free languages are equally well-behaved. Already the initial paper by Patnaik and Immerman showed that DynFO can maintain the membership problem for *Dyck languages* $D_k$, for $k \geq 1$, that is, the languages of well-balanced parentheses expressions with $k$ types of parentheses [20]. It was shown afterwards in [11, Theorem 4.1] that DynFO actually captures the membership problem for all context-free languages and that Dyck languages even do not require quantifiers in formulas (but functions in the auxiliary structure) [11, Proposition 4.4]. These results can easily be seen to apply to range queries as well. However, the dynamic program of [11, Theorem 4.1] uses 4-ary relations and three nested existential quantifiers, yielding work in the order of $n^7$.

In the following, we show that the membership problem for context-free languages is likely *not* solvable in DynFO with sublinear work, but that the Dyck language $D_1$ with one bracket type can be handled with polylogarithmic work for the membership problem and work $\mathcal{O}(n^\epsilon)$ for the range problem, and that for other Dyck languages these bounds hold with an additional linear factor $n$.

### 6.1 A conditional lower bound for context-free languages

Our conditional lower bound for context-free languages is based on a result from Abboud et al. [2] and the simple observation that the word problem for a language $L$ can be solved, given a dynamic program for its membership problem.

**Lemma 6.1.** *Let $L$ be a language. If* MEMBER$(L)$ *can be maintained in* DynFO *with work $f(n)$, then the word problem for $L$ can be decided sequentially in time $\mathcal{O}(n \cdot f(n))$.*

The announced lower bound is relative to the following conjecture [1].

*Conjecture 6.2 (k-Clique conjecture).* For any $\epsilon > 0$, and $k \geq 3$, $k$-Clique has no algorithm with time bound $\mathcal{O}(n^{(1-\epsilon)\frac{\omega}{3}k})$.

Here, $\omega$ is the matrix multiplication exponent [10,27], which is known to be smaller than 2.373 and believed to be exactly two [10,27].

In [2], the word problem for context-free languages was linked to the $k$-Clique problem as follows.

**Theorem 6.3 ([2, Theorem 1.1]).** *There is a context free grammar $G$ such that, if the word problem for $L(G)$ can be solved in time $T(n)$, $k$-Clique can be solved on $n$ node graphs in $\mathcal{O}(T(n^{\frac{k}{3}+1}))$ time, for any $k \geq 3$.*

Putting Lemma 6.1 and Theorem 6.3 together, we get the following result.

**Theorem 6.4.** *There is a context free grammar $G$ such that, if the membership problem for $L(G)$ can be solved by a* DynFO*-program with work $\mathcal{O}(n^{\omega-1-\epsilon})$, for some $\epsilon > 0$, then the $k$-Clique conjecture fails.*

The simple proofs of Lemma 6.1 and Theorem 6.4 are presented in the full version.

Thus, we can not reasonably expect any DynFO-programs for general context-free languages with considerable less work than $\mathcal{O}(n^{1.37})$ barring any breakthroughs for matrix multiplication. In fact, for "combinatorial DynFO-programs", an analogous reasoning yields a work lower bound of $\mathcal{O}(n^{2-\epsilon})$.

#### 6.2 On work-efficient dynamic programs for Dyck languages

We next turn to Dyck languages. Clearly, all Dyck languages are deterministic context-free, their word problem can therefore be solved in linear time, and thus the lower bound approach of the previous subsection does not work for them. We first present the DynFO-program with polylogarithmic work for the membership problem of $D_1$. It basically mimics the sequential algorithm from [8] that maintains $D_1$ sequentially in time $\mathcal{O}(\log n)$, per change and query operation.

**Theorem 6.5.** Member($D_1$) *can be maintained in* DynFO *with* $\mathcal{O}((\log n)^3)$ *work.*

*Proof sketch.* Let $\Sigma_1 = \{\langle, \rangle\}$ be the alphabet underlying $D_1$. The dynamic program uses an ordered binary tree $T$ such that each leaf corresponds to one position from left-to right. A parent node corresponds to the set of positions of its children. We assume for simplicity that the domain is $[n]$, for some number $n$ that is a power of 2. In a nutshell, the program maintains for each node $x$ of $T$ the numbers $\ell(x)$ and $r(x)$ that represent the number of unmatched closing and unmatched opening brackets of the string $\text{str}(x)$ corresponding to $x$ via the leaves of the induced subtree at $x$. E.g., if that string is $\rangle\langle\rangle\rangle\langle\langle\rangle$ for $x$, then $\ell(x) = 2$ and $r(x) = 1$. The overall string $w$ is in $D_1$ exactly if $r(\text{root}) = \ell(\text{root}) = 0$.

In the algorithm of [8], the functions $\ell$ and $r$ are updated in a bottom-up fashion. However, we will observe that they do not need to be updated sequentially in that fashion, but can be updated in parallel constant time. In the following, we describe how $\mathcal{P}$ can update $\ell(x)$ and $r(x)$ for all ancestor nodes $x$ of a position $p$, after a closing parenthesis $\rangle$ was inserted at $p$. Maintaining $\ell$ and $r$ for the other change operations is analogous.

There are two types of effects that an insertion of a closing parenthesis could have on $x$: either $\ell(x)$ is increased by one and $r(x)$ remains unchanged, or $r(x)$ is decreased by one and $\ell(x)$ remains unchanged. We denote these effects by the pairs $(+1, 0)$ and $(0, -1)$, respectively.

Table 1 shows how the effect of a change at a position $p$ below a node $x$ with children $y_1$ and $y_2$ relates to the effect at the affected child. This depends on whether $r(y_1) \leq \ell(y_2)$ and whether the affected child is $y_1$ or $y_2$. A closer inspection of Table 1 reveals a crucial observation: in the upper left and the lower right field of the table, the effect on $x$ is *independent* of the effect on the child (being it $y_1$ or $y_2$). That is, these cases induce an effect on $x$ independent of the children. We thus call these cases *effect-inducing*. In the other two fields, the

| | $p$ is in $\mathrm{str}(y_1)$ | $p$ is in $\mathrm{str}(y_2)$ |
|---|---|---|
| $r(y_1) \leq \ell(y_2)$ | $(+1,0) \to (+1,0)$<br>$(0,-1) \to (+1,0)$ | $(+1,0) \to (+1,0)$<br>$(0,-1) \to (0,-1)$ |
| $r(y_1) > \ell(y_2)$ | $(+1,0) \to (+1,0)$<br>$(0,-1) \to (0,-1)$ | $(+1,0) \to (0,-1)$<br>$(0,-1) \to (0,-1)$ |

**Table 1.** The effect on $x$ after a closing parenthesis was inserted at position $p$. The effects depend on the effect on the children $y_1$ and $y_2$ of $x$: for example, an entry '$(0,-1) \to (+1,0)$' in the column '$p$ is in $\mathrm{str}(y_1)$' means that if the change operation has effect $(0,-1)$ on $y_1$ then the change operation has effect $(+1,0)$ on $x$.

effect on $x$ depends on the effect at the child, but in the simplest possible way: they are just the same. That is the effect at the child is just adopted by $x$. We call these cases *effect-preserving*. To determine the effect at $x$ it is thus sufficient to identify the highest affected descendant node $z$ of $x$, where an effect-inducing case applies, such that for all intermediate nodes between $x$ and $z$ only effect-preserving cases apply.

Our dynamic program implements this idea. First it determines, for each ancestor $x$ of the change position $p$, whether it is effect-inducing and which effect is induced. Then it identifies, for each $x$, the node $z$ (represented by its height $i$ above $p$) as the unique effect-inducing node that has no effect-inducing node on its path to $x$. The node $z$ can be identified with work $\mathcal{O}((\log n)^2)$, as $z$ is one of at most $\log n$ many nodes on the path from $x$ to the leaf of $p$, and one needs to check that all nodes between $x$ and $z$ are effect-preserving. As the auxiliary relations need to be updated for $\log n$ many nodes, the overall work of $\mathcal{P}$ is $\mathcal{O}((\log n)^3)$. We refer to the full version for more details. □

**A work-efficient dynamic program for range queries for $D_1$ and $D_k$** Unfortunately, the program of Theorem 6.5 does not support range queries, since it seems that one would need to combine the unmatched parentheses of $\log n$ many nodes of the binary tree in the worst case. However, its idea can be combined with the idea of Proposition 5.2, yielding a program that maintains $\ell$ and $r$ for $\mathcal{O}(n^\epsilon)$ special intervals on a constant number of levels.

In fact, this approach even works for $D_k$ for $k > 1$. Indeed, with the help of $\ell$ and $r$, it is possible to identify for each position of an opening parenthesis the position of the corresponding closing parenthesis in $\mathcal{O}(1)$ parallel time with work $n^\epsilon$, and then one only needs to check that they match everywhere. The latter contributes an extra factor $\mathcal{O}(n)$ to the work, for $k > 1$, but can be skipped for $k = 1$.

**Theorem 6.6.** *For all $\epsilon > 0$, $k > 1$,*

*a)* RangeMember($D_1$) *can be maintained in* DynFO *with $\mathcal{O}(n^\epsilon)$ work, and*
*b)* RangeMember($D_k$) *can be maintained in* DynFO *with $\mathcal{O}(n^\epsilon)$ work per change operation and $\mathcal{O}(n^{1+\epsilon})$ work per query operation.*

*Proof sketch.* In the following we reuse the definition of *special intervals* from the proof of Proposition 5.2 as well as the definition of $\ell$ and $r$ from the proof of Proposition 6.5. We first describe a dynamic program for RANGEMEMBER($D_1$). It maintains $\ell$ and $r$ for all special intervals, which is clearly doable with $\mathcal{O}(n^\epsilon)$ work per change operation. Similar to the proof of Proposition 5.2, the two crucial observations (justified in the full version) are that (1) a range query can be answered with the help of a constant number of special intervals, and (2) the change operation affects only a bounded number of special intervals per level.

As stated before, the program for RANGEMEMBER($D_k$) also maintains $\ell$ and $r$, but it should be emphasised that also in the case of several parenthesis types, the definition of these functions ignores the bracket type. With that information it computes, for each opening bracket the position of its matching closing bracket, with the help of $\ell$ and $r$, and checks that they match. This can be done in parallel and with work $\mathcal{O}(n^\epsilon)$ per position. We refer to the full version for more details. □

**Moderately work-efficient dynamic programs for $D_k$** We now turn to the membership query for $D_k$ with $k > 1$. Again, our program basically mimics the sequential algorithm from [8] which heavily depends on the dynamic problem STRINGEQUALITY that asks whether two given strings are equal.

**Problem:** STRINGEQUALITY
**Input:** Two Sequences $u = u_0 \dots u_{n-1}$ and $v = v_0 \dots v_{n-1}$ of letters with $u_i, v_i \in \Sigma \cup \{\epsilon\}$
**Changes:** $\text{SET}_{x,\sigma}(i)$ for $\sigma \in \Sigma, x \in \{u, v\}$: Sets $x_i$ to $\sigma$, if $x_i = \epsilon$
$\text{RESET}_x(i)$ for $x \in \{u, v\}$: Sets $x_i$ to $\epsilon$
**Queries:** EQUALS: Is $u_0 \circ \dots \circ u_{n-1} = v_0 \circ \dots \circ v_{n-1}$?

It is easy to show that a linear amount of work is sufficient to maintain STRINGEQUALITY.

**Lemma 6.7.** STRINGEQUALITY *is in* DynFO *with work* $\mathcal{O}(n)$.

Because of the linear work bound for STRINGEQUALITY our dynamic program for MEMBER($D_k$) also has a linear factor in the work bound.

**Theorem 6.8.** MEMBER($D_k$) *is maintainable in* DynFO *with work* $\mathcal{O}(n \log n + (\log n)^3)$ *for every fixed* $k \in \mathbb{N}$.

*Proof sketch.* The program can be seen as an extension of the program for MEMBER($D_1$). As unmatched parentheses are no longer well-defined if we have more than one type of parenthesis the idea of [8] is to maintain the parentheses to the left and right that remain if we reduce the string by matching opening and closing parentheses regardless of their type. To be able to answer MEMBER($D_k$), the dynamic program maintains the unmatched parentheses for every node $x$ of a tree spanning the input word, and a bit $M(x)$ that indicates whether the types of the parentheses match properly.

How the unmatched parentheses can be maintained for a node $x$ after a change operation depends on the "segment" of $\text{str}(x)$ in which the change happened and in some cases reduces to finding a node $z$ with a local property on the path from $x$ to the leaf that corresponds to the changed position.

To update $M(x)$ for a node $x$ with children $y_1$ and $y_2$ the dynamic program compares the unmatched parentheses to the right of $y_1$ with the ones to the left of $y_2$ using StringEquality. We refer to the full version for more details. □

Maintaining string equality and membership in $D_k$ for $k > 1$ is even closer related which is stated in the following lemma.

**Lemma 6.9.** *a) If* StringEquality *can be maintained in* DynFO *with work* $W(n)$ *then* Member$(D_k)$ *can be maintained in* DynFO *with work* $\mathcal{O}(W(n) \cdot \log n + (\log n)^3)$*, for each* $k \geq 1$.
*b) If* Member$(D_k)$ *can be maintained in* DynFO *with work* $W(n)$ *for all* $k$*, then* StringEquality *can be maintained in* DynFO *with work* $\mathcal{O}(W(n))$.

### 7 Conclusion

In this paper we proposed a framework for studying the aspect of work for the dynamic, parallel complexity class DynFO. We established that all regular languages can be maintained in DynFO with $\mathcal{O}(n^\epsilon)$ work for all $\epsilon > 0$, and even with $\mathcal{O}(\log n)$ work for star-free regular languages. For context-free languages we argued that it will be hard to achieve work bounds lower than $\mathcal{O}(n^{\omega-1-\epsilon})$ in general, where $\omega$ is the matrix multiplication exponent. For the special case of Dyck languages $D_k$ we showed that $\mathcal{O}(n \cdot (\log n)^3)$ work suffices, which can be further reduced to $\mathcal{O}(\log^3 n)$ work for $D_1$. For range queries, dynamic programs with work $\mathcal{O}(n^{1+\epsilon})$ and $\mathcal{O}(n^\epsilon)$ exist, respectively.

We highlight some research directions. One direction is to improve the upper bounds on work obtained here. For instance, it would be interesting to know whether all regular languages can be maintained with polylog or even $\mathcal{O}(\log n)$ work and how close the lower bounds for context-free languages can be matched. Finding important subclasses of context-free languages for which polylogarithmic work suffices is another interesting question. Apart from string problems, many DynFO results concern problems on dynamic graphs, especially the reachability query [5]. How large is the work of the proposed dynamic programs, and are more work-efficient dynamic programs possible?

The latter question also leads to another research direction: to establish further lower bounds. The lower bounds obtained here are relative to strong conjectures. Absolute lower bounds are an interesting goal which seems in closer reach than lower bounds for DynFO without bounds on the work.
